# Mazabraud’s syndrome and thyroid cancer, a very rare and confusing association: a case report

**DOI:** 10.1186/s12902-015-0036-z

**Published:** 2015-08-06

**Authors:** Doina Piciu, Elena Barbus, Andra Piciu, Bogdan Fetica

**Affiliations:** Department of Endocrinology and Nuclear Medicine, Institute of Oncology “Prof. Dr. Ion Chiricuta” 34–36 Republicii, 400015 Cluj-Napoca, Romania; University of Medicine and Pharmacy “Iuliu Hatieganu” 6–8 V. Babes, 400012 Cluj-Napoca, Romania; Department of Oncology, Institute of Oncology “Prof. Dr. Ion Chiricuta” 34–36 Republicii, 400015 Cluj-Napoca, Romania; Department of Pathology, Institute of Oncology “Prof. Dr. Ion Chiricuta” 34–36 Republicii, 400015 Cluj-Napoca, Romania

**Keywords:** Mazabraud’s syndrome, Bone dysplasia, Myxoma, Thyroid cancer

## Abstract

**Background:**

Mazabraud’s syndrome is defined as the association between fibrous dysplasia and intramuscular myxomas. The syndrome was first described in 1967 and, up until now, less than 100 cases have been reported worldwide. Here we report the association between this rare syndrome and thyroid cancer. When a malignant disease occurs in a patient affected by this syndrome, the differential diagnosis between benign and malignant bone lesions should be undertaken carefully.

**Case presentation:**

We report the case of a 57-year-old Caucasian male, admitted for diffuse bone pain localized in the left leg and for the presence of an indolent, slow-growing mass in the left shoulder. The patient also presented with a thyroid nodule, highly suggestive of a malignancy. The radiologic examination showed multiple osteolytic lesions. The suspicion of multiple myeloma or bone metastases arising from a thyroid cancer was considered. Electrophoresis of proteins was negative and therefore excluded the diagnosis of multiple myeloma; the thyroid surgery was indicated. Thyroidectomy confirmed the papillary thyroid carcinoma, and the bone lesions were considered to be metastases from the thyroid cancer. After surgery, under thyroid-stimulated hormonal conditions, the patient underwent radioiodine therapy and a post-therapy radioiodine whole body scan. The lack of radioiodine uptake, both in the bone lesions and shoulder mass, suggested the possibility of less differentiated, non-avid radioiodine lesions, or the absence of any relation between pathologies. Considering the low level of the specific tumor marker, thyroglobulin, a bone biopsy and resection of the shoulder mass were indicated. The final diagnosis was intramuscular myxoma with polyostotic fibrous dysplasia in the deltoid muscle (Mazabraud’s syndrome). A completely incidental cerebral tumor lesion was also discovered.

**Conclusion:**

During the evolution of a malignant disease, Mazabraud’s syndrome, known as the association of intramuscular myxoma with fibrous dysplasia, should be considered in the differential diagnosis of bone metastasis. This is the first report in the literature of Mazabraud’s syndrome occurring in a patient with thyroid cancer.

## Background

In 1967, the French physician Mazabraud described the association of intramuscular myxoma with fibrous dysplasia for the first time, naming the syndrome after himself [1, 2]. Fibrous dysplasia is a benign lesion in intramedullary bone. It can be found in two forms: a polyostotic (disseminated bone lesions) and a monostotic form (unique bone lesion) [3]. Symptoms include localized tumoral and intermittent pain or even pathological fractures. The differential diagnosis includes neurofibromatosis, multiple myeloma, endochondromatosis, hyperparathyroidism and McCune–Albright syndrome [4]. Intramuscular myxomas represent benign mesenchymal tumors associated with the polyostotic form of fibrous dysplasia in most women.

The incidence of Mazabraud’s syndrome is <1/1,000,000 [4–6], with only 68–80 cases reported in the medical literature worldwide [1, 2]. The official Orphanet report, published in July 2015 confirms the presence of 54 cases published in the literature (ORPHA number 57782) [5]. The molecular diagnosis involves mutations in the GNAS1 gene, which encodes for an alpha subunit of a G protein [7].

Treatment of Mazabraud’s syndrome includes the specific treatment of the fibrous dysplasia when complications appear: pathological bone fractures treated using osteosynthesis, or when sarcoma transformation occurs, exceptionally, specific treatment for the malignant tumor. Intramuscular myxomas benefit from an excision and control of local and regional lesions.

We present the case of a 57-year-old Caucasian male with Mazabraud’s syndrome that was associated with a papillary thyroid carcinoma [8, 9]. A papillary thyroid cancer has been described in the literature, although in association with the McCune-Albright syndrome [7].

## Case presentation

A 57-year-old male was referred to the Institute of Oncology Cluj-Napoca for a second opinion, with suspicion of a multiple myeloma. The patient presented with diffuse post-traumatic pain in the left thorax and the left tibial diaphysis. The clinical exam revealed a tumor lesion localized in the left deltoid region and another one in the anterior cervical region, suggestive of thyroid nodules. Right lateral cervical enlarged lymph nodes were also described. Conventional radiological examination highlighted areas of osteolysis in the right IV^th^ costal arch and areas of osteolysis with ballooning in the left IV^th^, V^th^ and VII^th^ costal arches, as well as deformation and erosion of the cortical area of the bone.

**Fig. 1:**
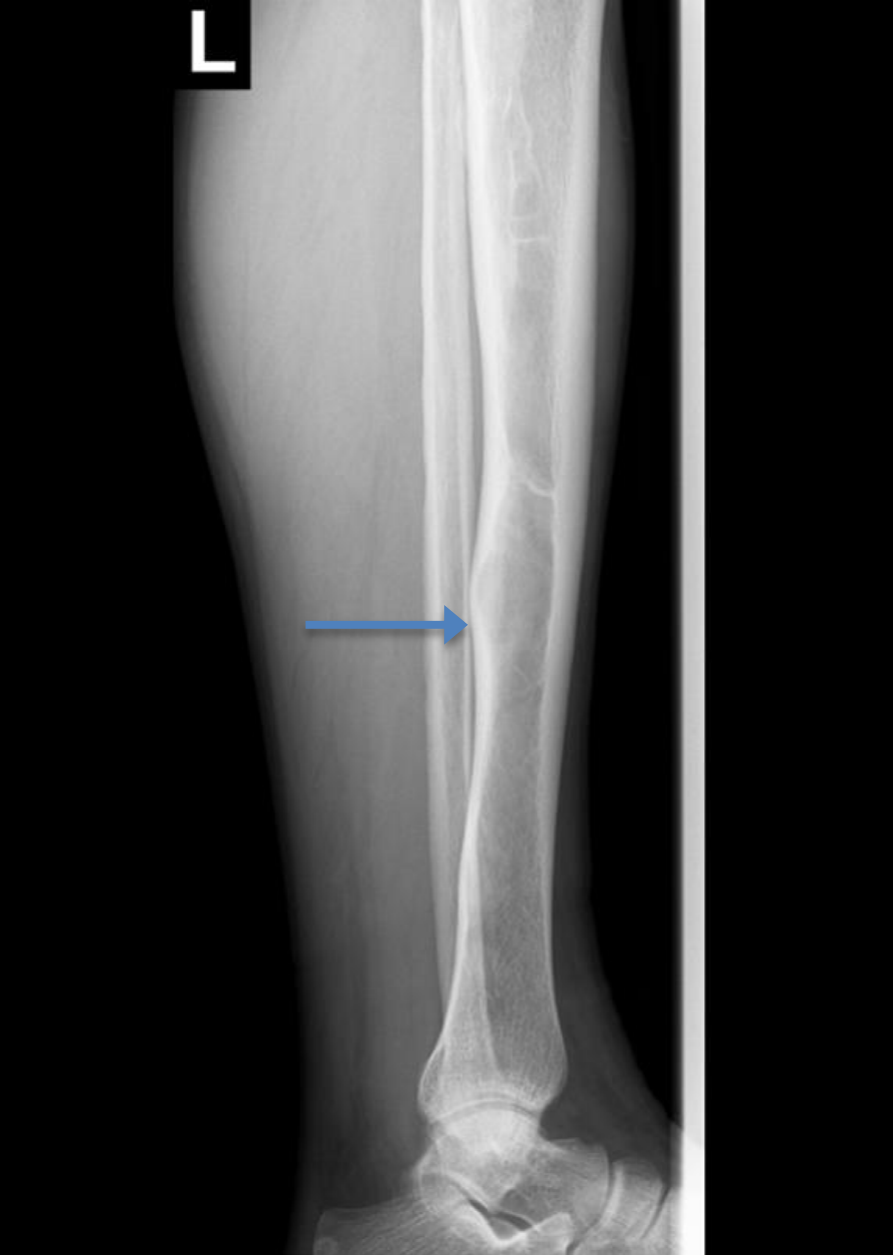
Tibial radiograph showing bone dysplasia in the middle and distal parts of the diaphysis

**Fig. 2:**
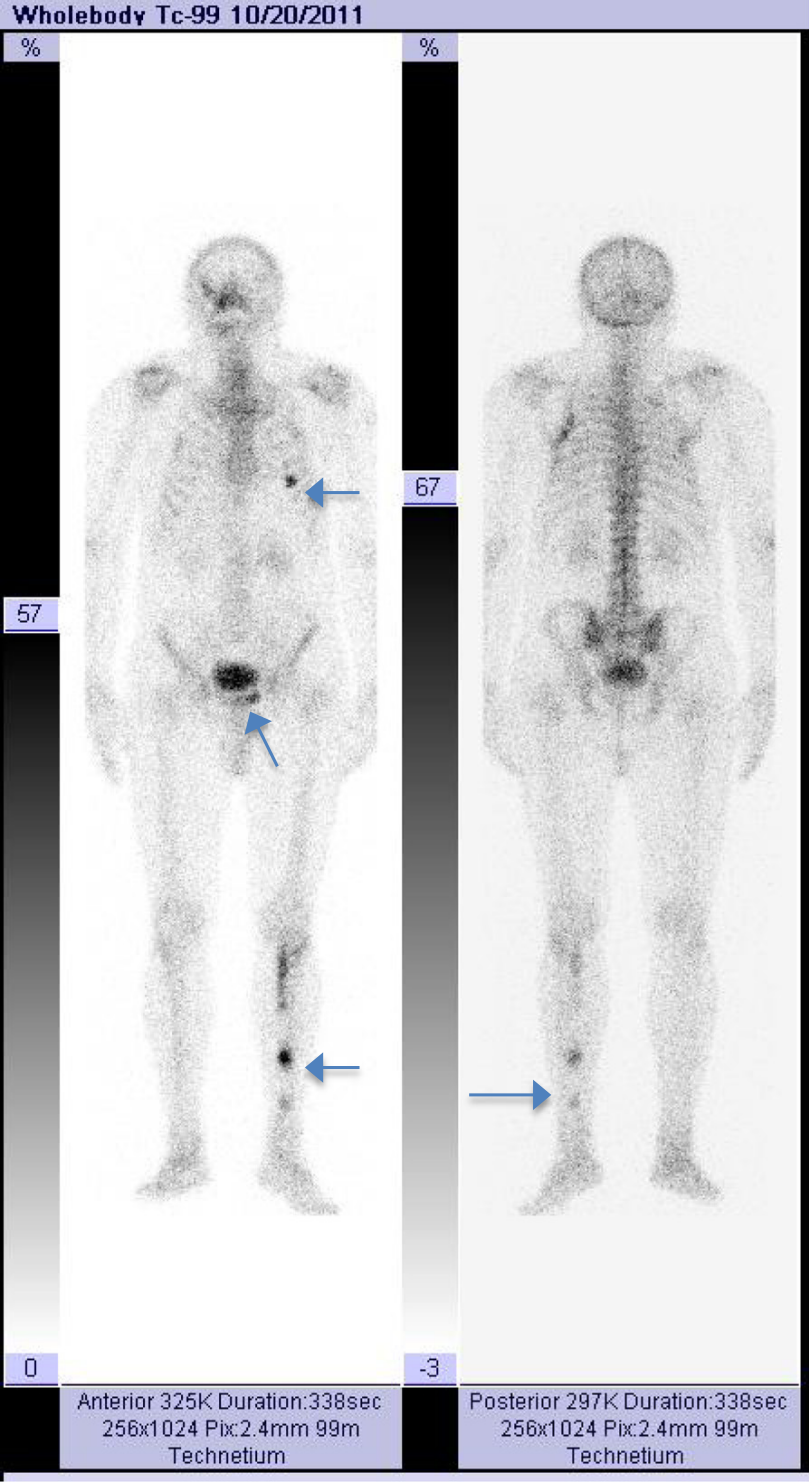
Whole body bone scintigraphy using Tc-99m MDP, showing pathologic uptake of tracer in thorax, pelvic area and left tibia

Radiography of the tibia revealed a left tibia diaphysis with an irregular aspect of the compact bone (Fig. 1). Suspicion of a multiple myeloma or metastases in the bones with an undetermined origin was raised. Immunofixation electrophoresis of proteins was negative and therefore excluded a diagnosis of multiple myeloma. The patient was directed to the nuclear medicine department for a bone scintigraphy, which revealed the pathological uptake of tracer at the level of the costal arches and in the left diaphysis of the tibia (Fig. 2).

**Fig. 3:**
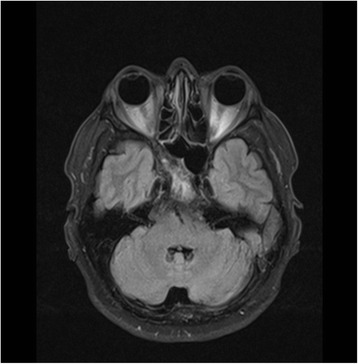
Brain MRI showing a tumor in the clivus and involvement of sphenoid bone

**Fig. 4:**
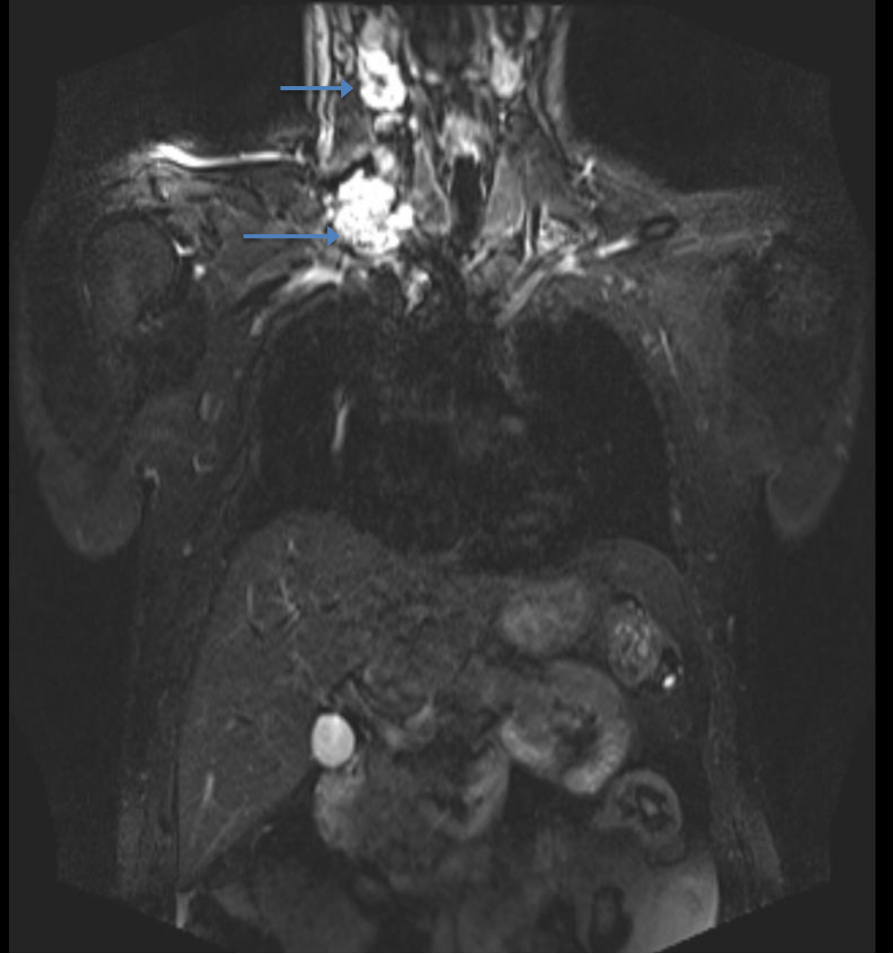
MRI of the cervical region showing the right thyroid lobe with a nodular structure and right cervical lymph nodes highly suspicious for malignancy

A blood sample was collected in order to determine the levels of tumor markers, which were at physiological normal levels: CA 19-9 = 25.2 U/mL (N.V. <27), CEA = 2.2 ng/mL (N.V. smokers <3.4; non-smokers <4.3), PSA = 1.31 ng/mL (N.V. <60 years old ≤3.1). Whole-body MRI showed multiple bone lesions localized at the level of the costal arches, left tibia, and left pubic branch, and an incidental tumor localized in the clivus that extended to the right sphenoid sinus (Fig. 3). In addition, there were multiple suspicious lymph nodes in the neck and in the right supraclavicular area and an inhomogeneous lesion in the right thyroid lobe (Fig. 4). The patient, at this point, was tentatively diagnosed to have thyroid cancer with bone and cutaneous metastases.

**Fig. 5:**
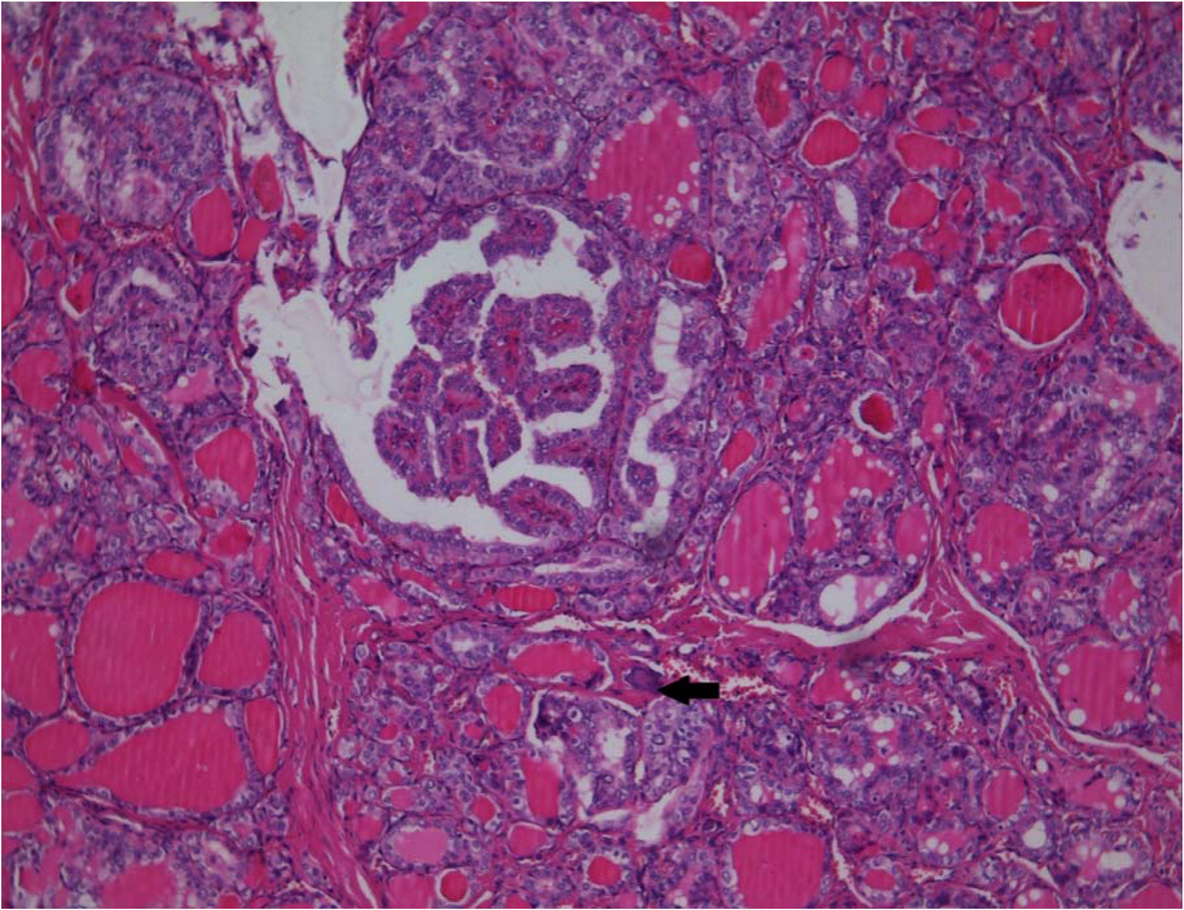
Histopathology of the thyroid gland showing a papillary thyroid carcinoma (H&E staining, 100× magnification). Tumor cells have specific nuclear changes and form follicles and papillary structures. A small psammomatous body is visible (indicated by the black arrow)

Blood samples were collected to determine thyroid stimulating hormone (TSH) and free-thyroxine-FT4 levels, which were found to be within normal limits. The thyroid peroxidase (TPO) test was also negative, thereby excluding a chronic lymphocytic thyroiditis. Calcium and parathyroid hormone (PTH) levels were also within normal limits, which excluded a parathyroid pathology, while a normal calcitonin level excluded a medullary thyroid carcinoma. Biopsy of one of the right cervical lymph nodes was performed and histology revealed a papillary thyroid cancer lymph node metastasis. Immunohistochemistry (IHC) of TTF-1 and thyroglobulin (TG) were positive in tumor cells. A total thyroidectomy and right selective lymphadenectomy were performed. This confirmed the diagnosis of a papillary thyroid carcinoma with lymph node metastasis (T3N1bMx stage IVA) (Fig. 5).

**Fig. 6:**
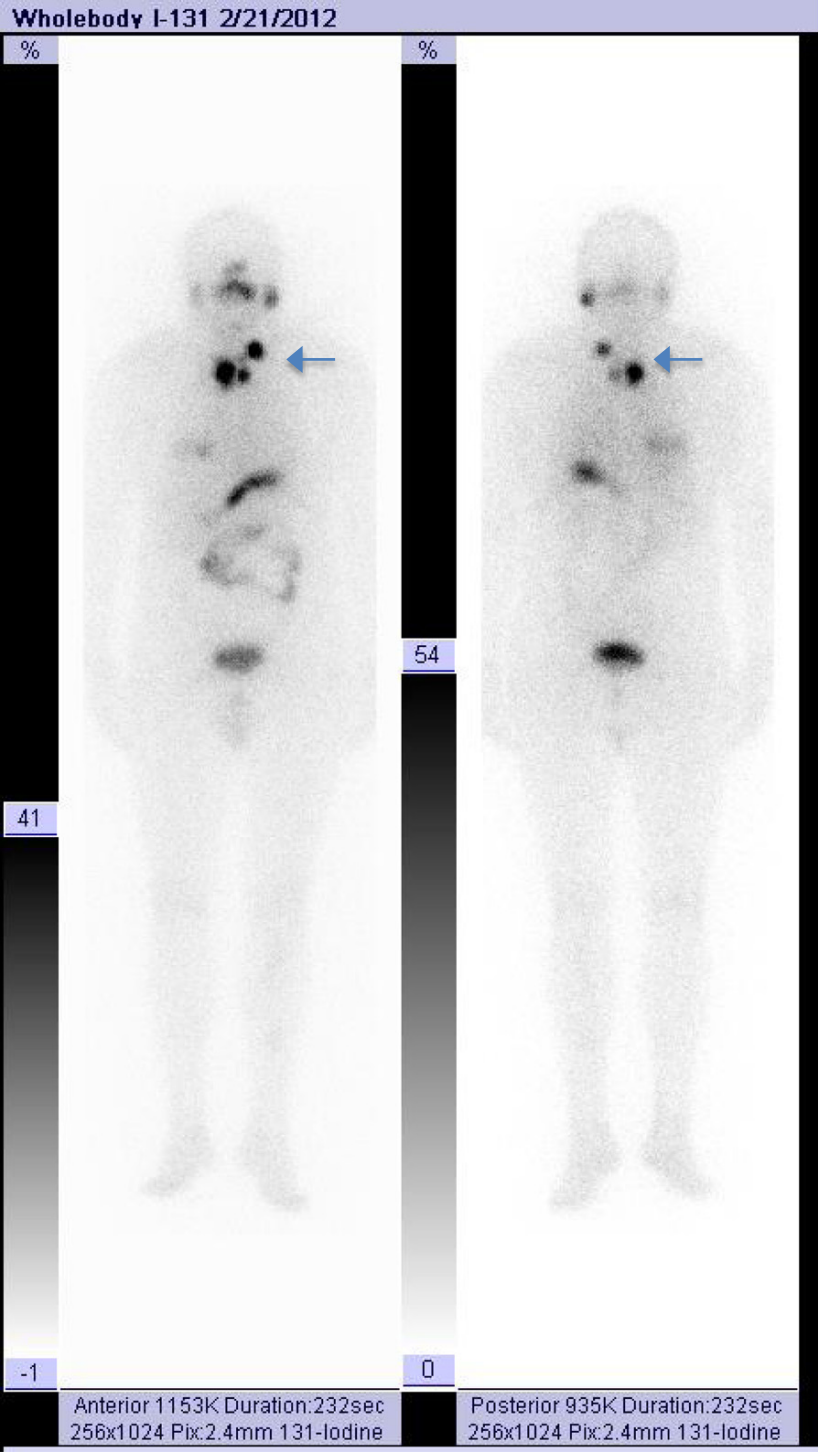
Whole body scan (WBS) at 48 h after radioiodine I-131 therapy showing pathologic uptake in the thyroid bed and left cervical lymph nodes

Radioiodine therapy was indicated and given at a total dose of 96 mCi (3.55 GBq). Figure 6 shows a whole body scan (WBS) at 48 h after the radioiodine cure and shows pathologic radioiodine uptake in the right thyroid bed, within the mediastinum area and in a left lateral cervical lymph node, but there was no uptake in the bone. There were at least three possibilities as to what the bone lesions were: 1) a less differentiated thyroid tumor, confirmed by the relatively low level of the specific tumor marker, TG = 28 ng/mL (N.V. <0.04 ng/mL in cured thyroid cancer patients, and negative for anti-TG antibodies) determined under stimulated TSH; 2) non-avid radioiodine lesions, confirmed by the lack of radioiodine uptake, both in the bone lesions and in the shoulder mass; and 3) the absence of any relation between the bone, the muscle pathologies and the thyroid cancer.

**Fig. 7:**
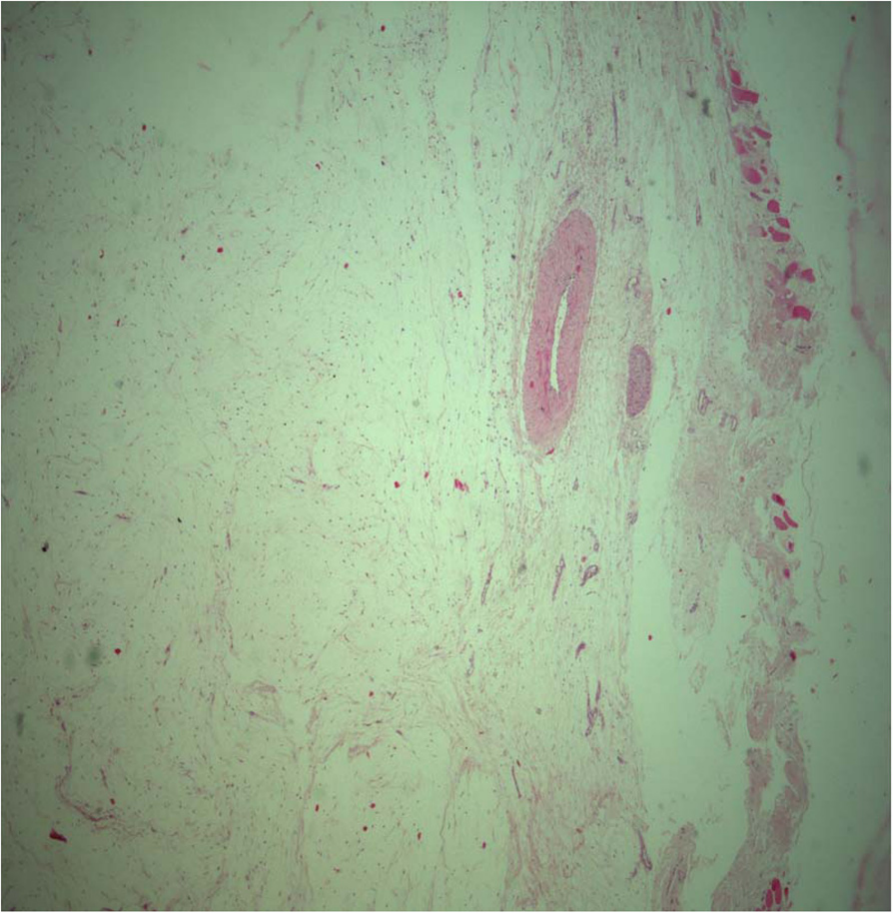
Histopathology of an intramuscular myxoma from a deltoid tumor (H&E staining, 25× magnification). Paucicellular tumor with myofibroblasts and fibroblasts embedded in a myxoid stroma. Mitotic activity is inconspicuous. Skeletal muscle fibers can be seen on the right

A biopsy of the shoulder tumor lesion was taken from the left deltoid area and the histology exam revealed a macroscopic white-translucent specimen of 3.5/3/2 cm, and of gelatinous consistency, without any areas of necrosis. Microscopic examination revealed tumor proliferation consisting of fusocellular and star cells, without any nuclear atypia and mitosis in a myxoid stroma. Tumor proliferation infiltrated into the rib muscles and resected margins came in contact with proliferating tumor cells. IHC was negative for S100, actin and desmin; the histologic aspect and IHC were specific for an intramuscular myxoma (Fig. 7). The association between the histology of the tumor and the bone lesions, with aspects of fibrous dysplasia, led to the diagnosis of Mazabraud’s syndrome. Biopsy of the tibia confirmed the diagnosis.

**Fig. 8:**
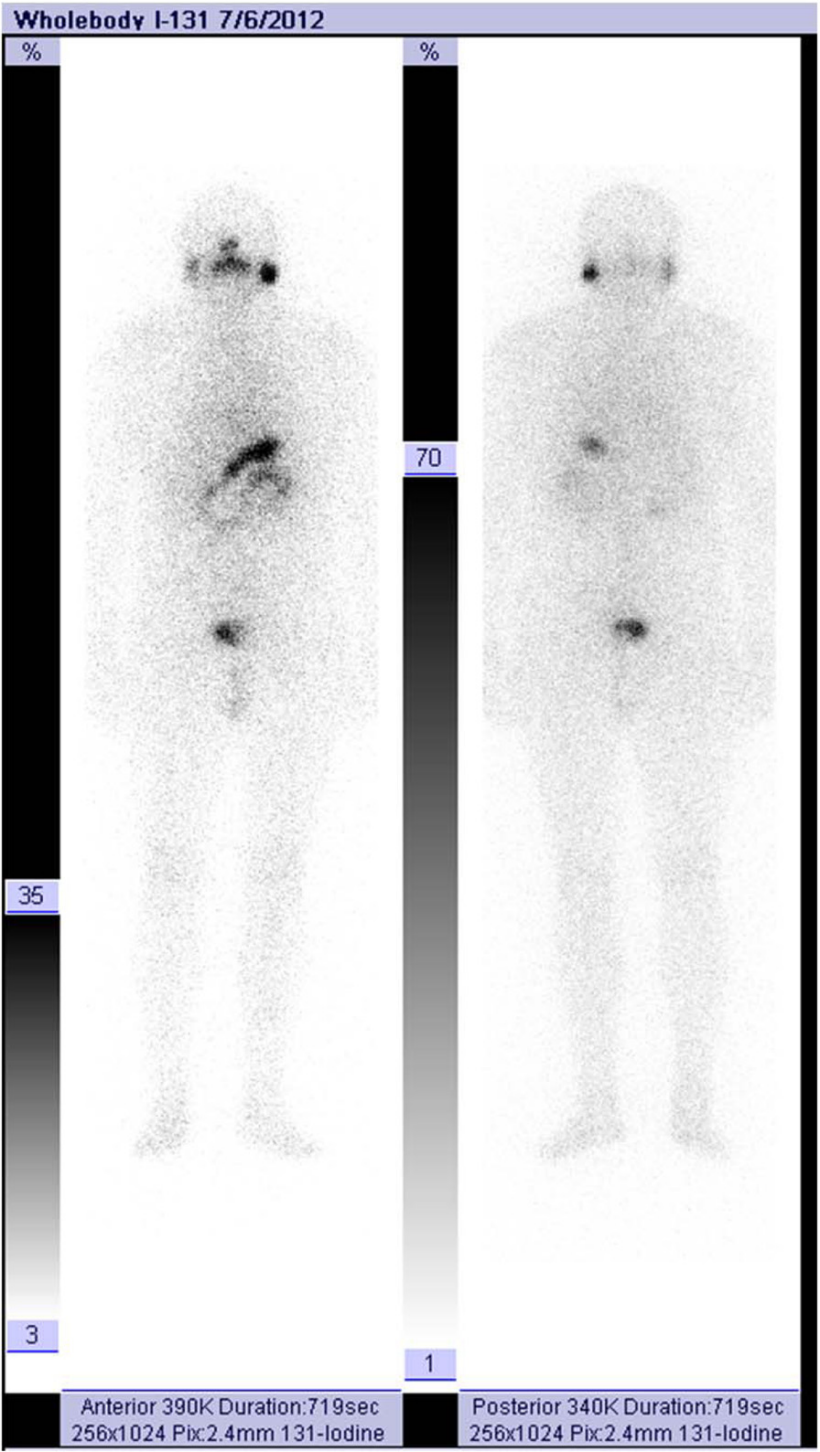
Negative whole body scan (WBS) 24 h after radioiodine I-131 showing no pathologic radioiodine uptake, thus confirming that the patient was cured of his/her thyroid cancer

At 2 years after surgery and irradiation, patient status revealed that: clinical tests and thyroid ultrasound were negative, antithyroglobulin (anti-TG) was <10 UI/mL (N.V. ≤115), TSH was 21.91 μUI/mL, TG was 0.82 ng/mL (N.V. athyroid patients <0.04) and I-131 WBS was negative (Fig. 8). The patient was therefore considered cured of his thyroid cancer and yearly follow-up was implemented; the bone lesions attributed to osseous dysplasia were stable.

**Fig. 9:**
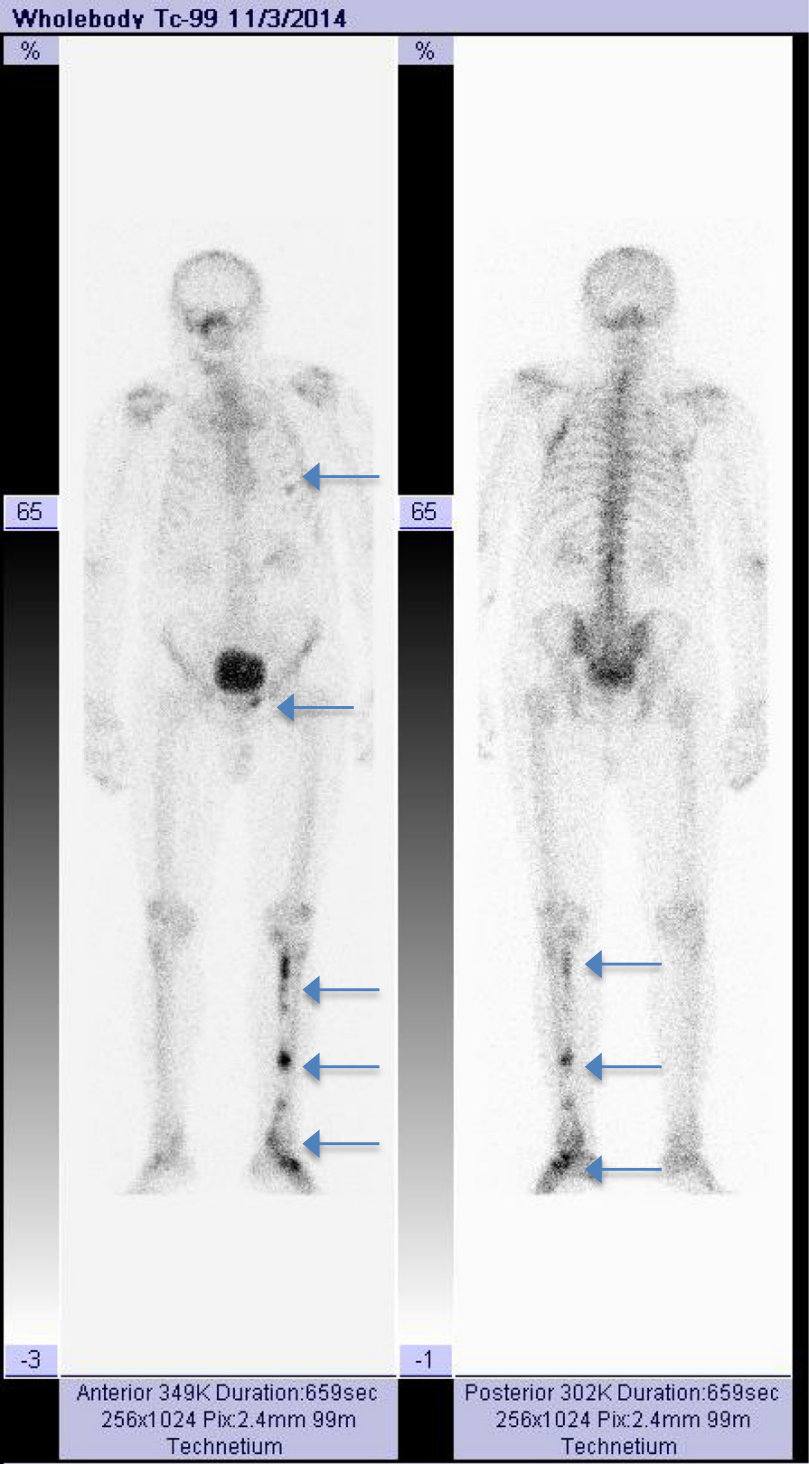
Whole body bone scintigraphy with Tc-99m MDP at 3 years after the initial diagnosis, showing pathologic uptake of the tracer in thorax and left tibia, suggesting evolution of the disease in the left leg (indicated by arrows)

During the follow-up at 3 years after the initial diagnosis of Mazabraud’s syndrome a bone scintigraphy was performed and confirmed the bone tumor lesions already known, as well as a new lesion in the left ankle articulation, further corroborating the diagnosis of Mazabraud’s syndrome (Fig. 9). No clinical signs of relapse were noted in the left deltoid area. No genetic testing for mutations in the GNAS1 gene was performed because it was not available nationally. The cerebral lesion described in the MRI was not biopsied, being refused by patient, and was therefore strictly monitored by the neurosurgery specialist.

## Discussion

The medical case presented is typical of Mazabraud’s syndrome, as described in the literature, however, the association with a malignant disease, seriously complicated the medical diagnosis and treatment.

Fibrous dysplasia has no predilection for the axial skeleton or the appendicular one. Statistics show that fibrous dysplasia has an incidence of 7 % of all benign bone lesions [9]. In the case presented, the polyostotic form was predominant, with lesions being situated in the diaphysis-metaphysis region, pelvis and the costal arches, which occurs with a frequency of 15 % of all lesions. The trigger for this type of lesion is a genetic mutation in the GNAS1 gene situated on chromosome 20q that encodes the alpha subunit of a G protein, which plays an important role in cell proliferation [8]. Fibrous dysplasia can also occur in the McCune–Albright syndrome, in which café-au-lait spots and endocrine disorders appear (nodular goiter, diabetes, early puberty and others) [4].

There are usually multiple intramuscular myxomas that occur in Mazabraud’s syndrome that appear before any bone lesion [10]. The mean age at which Mazabraud’s syndrome is diagnosed is 47 years, and the disease is predominant in the female population [8]. The most frequent pathologies associated with Mazabraud’s syndrome are benign thyroid diseases and early puberty. When a malignant tumor such as thyroid cancer is suspected, the presence of any other tumors in the brain, bones or soft tissues should be considered as potential metastases, unless other pathologies are ruled out. In this particular case, although a possible association between the two entities of myxoma and osseous dysplasia in Mazabraud’s syndrome is uncommon, it allowed the clinician to select the correct radical therapeutic strategy to treat the thyroid pathology.

The imaging techniques used for the diagnosis of Mazabraud’s syndrome involved conventional radiology to outline the bone lesions from the fibrous dysplasia using the “ground-glass” aspect and presenting areas of bone sclerosis [2]. Bone scintigraphy was indicated to outline the polyostotic form and the implication of multiple bone structures. MRI was used to detect muscle tumor lesions and to find new lesions that were not clinically evident [1]. Microscopy of the intramuscular myxoma was specific: hypocellularity, with a myxoid stroma, stellar aspect of the cells and the absence of nuclear atypia and of the necrosis [2].

There is no specific treatment for Mazabraud’s syndrome. A follow-up of the bone pathology was indicated in order to detect a possible malignant transformation. Intramuscular myxomas can be excised when they cause local pain [9]. The association with a malignant disease is very challenging, especially if the genetic test is missing.

In the present clinical case, the specific uptake of radioiodine into only normal and tumoral thyroid cells during the WBS was the main reason for understanding the non-thyroid origin of the bone lesions. The chance of this case was the good prognostic of the malignant disease, which was considered for radical treatment. The limited information about Mazabraud’s syndrome and its low incidence might lead to the use of the wrong therapeutic strategy if other bone metastatic malignant diseases are involved.

## Conclusions

Mazabraud’s syndrome continues to be a very rare disease, with <100 cases worldwide. Therefore, in assessing a bone lesion associated with a malignant disease, we should be warned about its features, considering that the confusing clinical presentation might result in inappropriate staging and therapy for a cancer patient.

## Consent

Written informed consent was obtained from the patient for publication of this Case report and any accompanying images. A copy of the written consent is available for review by the Editor of this journal.
